# *UME6* Is Involved in the Suppression of Basal Transcription of ABC Transporters and Drug Resistance in the ρ^+^ Cells of *Saccharomyces cerevisiae*

**DOI:** 10.3390/microorganisms10030601

**Published:** 2022-03-10

**Authors:** Yoichi Yamada

**Affiliations:** Faculty of Biological Science and Technology, Institute of Science and Engineering, Kanazawa University, Kanazawa 920-1192, Japan; yamada-y@se.kanazawa-u.ac.jp; Tel.: +81-76-234-4898; Fax: +81-76-234-4900

**Keywords:** *Saccharomyces cerevisiae*, *UME6*, *RPD3*, *PDR5*, pleiotropic drug resistance, ρ^+^ cells

## Abstract

In *Saccharomyces* *cerevisiae*, the Rpd3L complex contains a histone deacetylase, Rpd3, and the DNA binding proteins, Ume6 and Ash1, and acts as a transcriptional repressor or activator. We previously showed that *RPD3* and *UME6* are required for the activation of *PDR5*, which encodes a major efflux pump, and pleiotropic drug resistance (PDR) in ρ^0/−^ cells, which lack mitochondrial DNA. However, there are inconsistent reports regarding whether *RPD3* and *UME6* are required for Pdr5-mediated PDR in ρ^+^ cells with mitochondrial DNA. Since *PDR5* expression or PDR in the ρ^+^ cells of the *rpd3*Δ and *ume6*Δ mutants have primarily been examined using fermentable media, mixed cultures of ρ^+^ and ρ^0/−^ cells could be used. Therefore, we examined whether *RPD3* and *UME6* are required for basal and drug-induced *PDR5* transcription and PDR in ρ^+^ cells using fermentable and nonfermentable media. *UME6* suppresses the basal transcription levels of the ABC transporters, including *PDR5*, and drug resistance in ρ^+^ cells independent of the carbon source used in the growth medium. In contrast, *RPD3* is required for drug resistance but did not interfere with the basal *PDR5* mRNA levels. *UME6* is also required for the cycloheximide-induced transcription of *PDR5* in nonfermentable media but not in fermentable media.

## 1. Introduction

In the yeast *Saccharomyces cerevisiae,* ATP-binding cassette (ABC) transporters, such as Pdr5, Snq2, and Yor1, efflux a variety of functionally and structurally unrelated compounds, such as fluconazole and cycloheximide from cells [1,2,3,4,5]. The overexpression of the ABC transporters causes multidrug resistance, which is referred to as pleiotropic drug resistance (PDR) in *S. cerevisiae* [6,7]. The ABC transporters *PDR5*, *SNQ2*, *YOR1*, *PDR10*, and *PDR15* are regulated by the paralogous Zn_2_Cys_6_ transcription factors, Pdr1 and/or Pdr3 [1,8,9]. Pdr1 or Pdr3 recognizes the pleiotropic drug response elements (PDRE) DNA consensus motifs in the promoter regions of *PDR5*, *SNQ2*, *YOR1*, *PDR10*, and *PDR15* [9,10,11,12]. *PDR3* also has two PDREs in its promoter region that are recognized by Pdr3 and Pdr1 [13]. Thus, *PDR3* is subject to positive transcriptional autoregulation by Pdr3 and transcriptional regulation by Pdr1, via these two PDREs [13]. The basal expression levels of *PDR5* and *SNQ2* and PDR are reduced by *PDR1* deletion in ρ^+^ cells, which contains mitochondrial DNA, compared to *PDR3* deletion [10,12]. The disruption of *PDR1* and *PDR3* substantially reduces the basal expression of their target genes, including *PDR5*, and causes hypersensitivity to multiple drugs compared with the disruption of either *PDR1* or *PDR3* [10,12]. Thus, *PDR1* and *PDR3* have functionally overlapping roles in PDR.

Gain-of-function mutations, such as pdr1-3 and pdr3-7 in Pdr1 and Pdr3, lead to the overexpression of *PDR3*, *PDR5*, *SNQ2*, *YOR1*, *PDR10*, and *PDR15,* and thereby induce the PDR [6,12,14,15,16]. This suggests that the exogenous compounds and drugs lead to the release of Pdr1 and Pdr3 from certain negative regulators because Pdr1 and Pdr3 directly bind a variety of exogenous compounds and drugs [17]. On the other hand, cycloheximide, fluphenazine, or cantharidin induces the transcription of *PDR1* and *PDR3* [18,19,20]. However, the physiological significance underlying this regulation remains unclear. Interestingly, overproduction of either Pdr1 or Pdr3 in the absence of exogenous drugs can lead to the induction of target gene expression [6,10]. In addition, the *PDR5, SNQ2, YOR1, PDR10,* or *PDR15* mRNA levels were found to be upregulated by the compounds such as cycloheximide, benomyl, fluphenazine, 4-nitroquinolineN-oxide, cantharidin, 2,4-dichlorophenol, or polyoxyethylene-9-laurylether [18,19,20,21,22]. Pdr1 and Pdr3 are also responsible for the transcriptional induction of the ABC transporters such as *PDR5, SNQ2*, and *YOR1* after exposure to drugs or chemical compounds [18,22,23]. The retrograde signaling pathway is strongly activated in ρ^0^ cells lacking mitochondrial DNA in *S. cerevisiae* and *Candida glabrata*, resulting in the enhanced expression of multidrug resistance genes, including *PDR5* and *CgCdr1*, and the elevated PDR. Notably, Pdr3, but not Pdr1, is required for the induction of *PDR5* expression in ρ^0^ cells [10,24].

In *S. cerevisiae*, there are two Rpd3 complexes, Rpd3S (0.6 MDa) and Rpd3L (1.2 MDa). Both complexes share Rpd3, Sin3, and Ume1 [25]. Rpd3 is a histone deacetylase that participates in chromatin remodeling and transcriptional repression [26,27]. Interestingly, Rpd3 is also required for Hsp90-dependent antifungal drug resistance. The Rpd3L complex contains the DNA binding proteins Ume6 and Ash1 and is thereby recruited by the proteins at the target gene promoters [12,28]. For example, the Rpd3L and Isw2 chromatin remodeling complexes are recruited by Ume6 at early meiotic gene promoters to repress their expression [27,29]. Alternatively, the Rpd3L complex is targeted to the *HO* promoter by Ash1 to specifically inhibit its expression [25]. In addition to transcriptional repression, Rpd3 is also required for transcriptional activation of osmoresponsive [30] and the DNA damage-inducible genes [31]. 

We previously showed that *RPD3* and *UME6* are required for the activation of *PDR5* transcription and drug resistance by retrograde signaling in the ρ^0^ cells of *S. cerevisiae* [32]. On the other hand, Borecka-Melkusova et al. showed that the ρ^+^ cells in the *rpd3*Δ, but not the *ume6*Δ and *ash1*Δ strains, displayed sensitivity to cycloheximide at the minimum inhibitory concentration [33]. They also reported that disruption of *RPD3* reduces basal *PDR5* transcription levels in ρ^+^ cells [33]. However, Robbins et al. reported that the increased azole susceptibility observed in ρ^+^ cells of the *rpd3*Δ strains of *Candida albicans* and *S. cerevisiae* is not due to reduced *PDR5* mRNA levels but instead due to diminished Hsp90-dependent antifungal drug resistance [34]. In addition, Yibmantasiri et al. reported that the deletion of *UME6* in ρ^+^ cells conferred sensitivity to atorvastatin, cycloheximide, and benomyl but not to ketoconazole, fluconazole, and oligomycin via spot dilution assay [35]. The authors also reported that deletion of *UME6* does not reduce Pdr5 expression by western blot analysis [35]. Thus, there are discrepancies regarding whether *RPD3* and *UME6* are required for *PDR5*-mediated PDR in ρ^+^ cells.

However, in the above studies, ρ^+^ cells grown in yeast extract peptone dextrose (YPD) culture medium containing glucose as the carbon source are used when drug susceptibility and basal *PDR5* expression were examined. Since ρ^+^ cells grown in YPD medium spontaneously generate ρ^0/−^ cells that lack mitochondrial DNA at a rate of 0.1–1% per generation, the above studies could have used mixed cultures of ρ^+^ and ρ^0/−^ cells [36,37]. ρ^0/−^ cells can grow on YPD culture medium containing glucose as a fermentable carbon source but cannot grow on yeast extract peptone glycerol (YPG) medium containing glycerol as a nonfermentable carbon source. Thus, we can examine the effect of *RPD3* and *UME6* deletion on *PDR5* transcription and PDR in pure ρ^+^ cells grown on YPG culture medium. Therefore, in this study, we examined whether *RPD3* and *UME6* are required for basal and drug-induced *PDR5* transcription and drug resistance in the ρ^+^ cells of *S. cerevisiae* grown in YPG medium. Furthermore, the results obtained in YPG medium were compared with those obtained from ρ^+^ cells grown in YPD medium. As a result, we show that *UME6* suppresses the basal transcription levels of the ABC transporters, including *PDR5*, and drug resistance in ρ^+^ cells independent of the carbon source used in the growth medium. In contrast, we also show that *RPD3* is required for drug resistance but did not interfere with the basal *PDR5* mRNA levels.

## 2. Materials and Methods

### 2.1. Yeast Strains and Media

The FY1679-28C (MATa, ura3-52, leu2-D1, trp1-D63, his3-D200, GAL2+) strain was used as the wild-type strain [38]. To construct its derivatives with *UME6*, *RPD3*, *PDR3*, *ASH1*, or *GAT3* deletions, open reading frames of the genes were replaced with *KanMX* or *bleMX6* gene cassettes by PCR-mediated one-step gene disruption in the FY1679-28C background [39]. The strains described above were grown on glycerol-rich YPG agar plates (2% glycerol, 1% yeast extract, 2% bactopeptone, 2% agar) to eliminate ρ^0^ cells and obtain ρ^+^ cells. 

Yeast cells were grown in YPD medium (2% glucose, 1% yeast extract, 2% bactopeptone) or YPG medium (2% glycerol, 1% yeast extract, 2% bactopeptone) at 30 °C with shaking.

### 2.2. Spot Dilution Assay

The relative resistance of each yeast strain to fluconazole or cycloheximide was estimated with a spot dilution assay using YPG or YPD media [40,41]. ρ^+^ cells from each yeast strain were aerobically grown to the logarithmic phase (an OD_600_ of 0.6–0.9) at 30 °C in YPG or YPD media in triplicate. Five microliters of 10-fold serial dilutions of the YPG cultures containing the same number of cells was spotted on YPG plates with or without 3 μg/mL fluconazole (Nacalai Tesque) (or 0.1 μg/mL cycloheximide (Wako)) and incubated at 30 °C for 21 days. Five microliters of 10-fold serial dilutions of the YPD cultures containing the same number of cells was spotted on YPD plates with or not without 30 μg/mL fluconazole (Nacalai Tesque) (or 0.5 μg/mL cycloheximide (Wako)) and incubated at 30 °C for 21 days.

### 2.3. Cocultivation of Two Gene Deletion Mutants Replaced with KanMX or bleMX6 Gene Cassettes

One gene deletion mutant replaced with the *KanMX* gene cassette was cocultivated with the other gene deletion mutant that had been replaced with the *bleMX6* gene cassette [32]. ρ^+^ cells from each mutant corresponding to 0.1 OD_600_ initial concentration were cocultivated in 10 mL of YPG or YPD media with or without 30 μg/mL fluconazole (or 0.5 μg/mL cycloheximide) [32]. The aliquots of the coculture were recovered immediately before drugs addition (at 0 min) and at various times after drug addition and spread on the YPD plates containing G418 or Zeocin [32,42]. The viability of each strain at each time point was estimated from the colony numbers on the G418 and Zeocin plates [32,42]. 

### 2.4. RNA Extraction from the ρ^+^ Cells in Each Strain Grown to the Logarithmic Phase

ρ^+^ cells from each yeast strain were grown to the logarithmic phase (an OD_600_ of 7–9) in YPG or YPD media in duplicate. The cultures were diluted to an OD_600_ of 0.1 and grown to an OD_600_ of 0.4–0.8 [32,43,44]. Aliquots of the cultures were recovered. The cells in the aliquots above were pelleted, washed, frozen at –80 °C, and used to extract total RNA [32,43,44]. Total RNA was isolated from the yeast cells using a NucleoSpin RNA kit (TaKaRa) according to the manufacturer’s protocol.

### 2.5. RNA Extraction from ρ^+^ Cells before and after Cycloheximide Exposure

ρ^+^ cells from the wild-type and *ume6*∆꞉꞉*bleMX6* strains were grown to an OD_600_ of 7–9 in YPG or YPD media, diluted to an OD_600_ of 0.1, and grown to an OD_600_ of 0.4–0.8 in duplicate [32,43,44]. Aliquots of each duplicate were harvested just prior to the addition of cycloheximide to the medium. The duplicates were grown for 45 min or 90 min at 30 °C after exposure to cycloheximide (0.2 μg/mL). Aliquots of each duplicate were recovered at 45 min or 90 min after the addition of cycloheximide. The cells in the aliquots above were pelleted, washed, frozen at –80 °C, and used to extract total RNA [32,43,44]. Total RNA was isolated from the yeast cells before and after exposure to cycloheximide using a NucleoSpin RNA Plus kit (TaKaRa) according to the manufacturer’s protocol. 

### 2.6. Real-Time RT–PCR

Reverse transcription of total RNA was performed using the PrimeScript 1st strand cDNA Synthesis Kit (TaKaRa). SYBR Green qRT-PCR for cDNA from the individual duplicate samples was performed using the TB Green^®^ Premix Ex Taq II (TaKaRa) in a Step One Real-time PCR system (Applied Biosystems). A minus reverse transcriptase control was used as the negative control. Serial dilutions of control cDNA were prepared to produce a standard curve for each primer pair. The primers used for qRT-PCR are listed in Table S1. The transcription levels of each target gene were measured by qRT-PCR and normalized to *ACT1,* which was used as an endogenous control. The normalized levels are shown relative to samples from the wild-type or cycloheximide-untreated wild-type strains, which were set to 1.

### 2.7. Statistical Analysis

The survival rate of the *ume6*∆ strain at each time point in Figure 2 was normalized to that at 0 h. A paired *t*-test was used for statistical analysis in Figure 2. An unpaired Student’s *t*-test was used for statistical analysis in Figures 3 and 4. Statistical significance was indicated as *p* < 0.05.

## 3. Results

### 3.1. UME6 Acts as a Negative Regulator to Fluconazole- and Cycloheximide-Resistance in ρ^+^ Cells, While RPD3 Acts as a Positive Regulator

**Figure 1:**
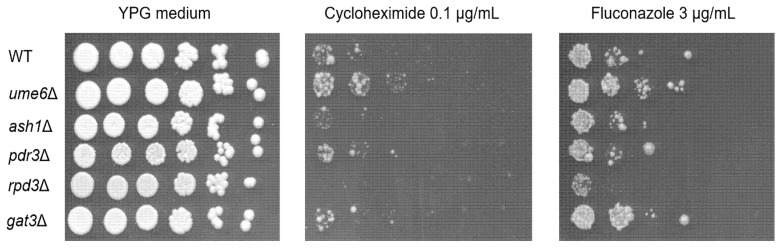
The ρ^+^ cells of the *ume6*∆ mutant are more tolerant to fluconazole and cycloheximide than those of the wild type on YPG plates. Fluconazole and cycloheximide resistance of ρ^+^ cells of the wild-type strain (FY1679-28C) and its derivative strains, *ume6*∆꞉꞉*bleMX6*, *ash1*∆꞉꞉*bleMX6*, *pdr3*∆꞉꞉*bleMX6*, and *rpd3*∆꞉꞉*bleMX6*, and *gat3*∆꞉꞉*bleMX6*, were determined by spot dilution assay in YPG plates with or without 3 μg/mL fluconazole (or 0.1 μg/mL cycloheximide).

To examine whether *RPD3* and *UME6* are responsible for PDR in ρ^+^ cells of *S. cerevisiae*, a spot dilution assay using YPG medium containing the nonfermentable carbon source glycerol was carried out to determine the resistance or susceptibility in the ρ^+^ cells of the *rpd3*∆ and *ume6*∆ mutants to the PDR substrates fluconazole and cycloheximide. ρ^+^ cells of the wild-type strain required a lower concentration of fluconazole and cycloheximide for growth inhibition in YPG medium than in YPD medium, which is consistent with previous reports [45]. The ρ^+^ cells of the *ume6*∆꞉꞉*bleMX6* mutant were slightly more tolerant to fluconazole and cycloheximide than those in the wild-type, *ash1*∆꞉꞉*bleMX6*, *pdr3*∆꞉꞉*bleMX6*, and *gat3*∆꞉꞉*bleMX6* strains (Figure 1). However, the ρ^+^ cells of the *rpd3*∆ mutant were more susceptible to fluconazole and cycloheximide than those in the wild-type, *ume6*∆꞉꞉*bleMX6, ash1*∆꞉꞉*bleMX6*, *pdr3*∆꞉꞉*bleMX6*, and *gat3*∆꞉꞉*bleMX6* strains (Figure 1).

The oligomycin susceptibility of the yeasts was tested on nonfermentable media like YPG medium [3,46]. However, Jensen et al. reported impaired endoplasmic reticulum (ER) to Golgi trafficking of Pdr5 in the *rpd3*Δ strain of ρ^+^ *S. cerevisiae* in nonfermentable medium with glycerol and ethanol as the carbon sources but not in YPD medium [45]. In addition, the *PDR5* mRNA levels are downregulated in the ρ^+^ cells of *S. cerevisiae* limited for glucose [8]. These reports indicate that the expression of the regulatory machinery of *PDR5* in ρ^+^ cells could be different between YPG and YPD cultures. Therefore, to examine whether *RPD3* and *UME6* are involved in PDR in ρ^+^ cells cultured in YPD medium in the same way as that in YPG medium, a similar spot dilution assay as that described above was conducted in YPD plates with or without 30 μg/mL fluconazole (or 0.5 μg/mL cycloheximide). As a result, there were no significant differences in fluconazole and cycloheximide resistance between the wild-type, *ume6*∆꞉꞉*bleMX6*, *ash1*∆꞉꞉*bleMX6*, *pdr3*∆꞉꞉*bleMX6*, and *gat3*∆꞉꞉*bleMX6* strains in YPD plates (Figure S1). However, significantly higher susceptibilities to fluconazole and cycloheximide were found in the *rpd3*∆ mutant than those found in the wild-type strain (Figure S1). The cycloheximide insusceptibility found in the ρ^+^ cells of the *ume6*∆ and *ash1*∆ mutants in YPD medium was consistent with reports by Borecka-Melkusova et al. [33]. On the other hand, fluconazole insusceptibility, but not cycloheximide insusceptibility, in the ρ^+^ cells of the *ume6*∆ mutant in YPD was consistent with reports by Yibmantasiri et al. [35].

**Figure 2:**
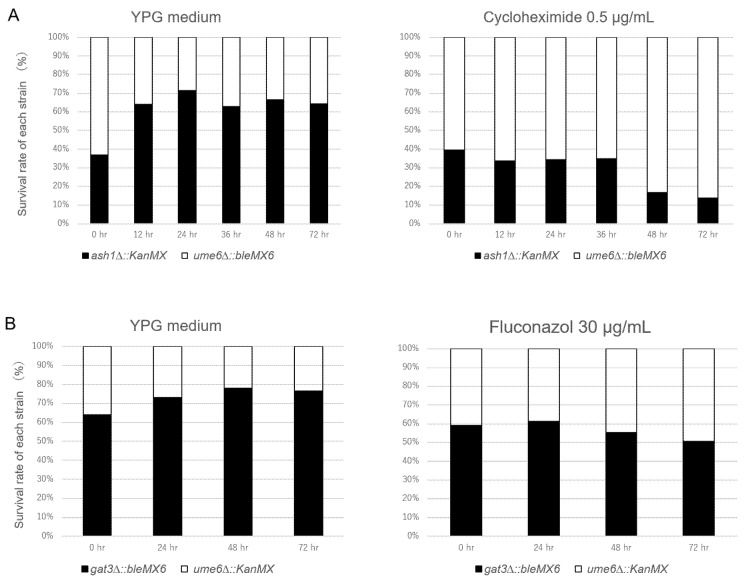

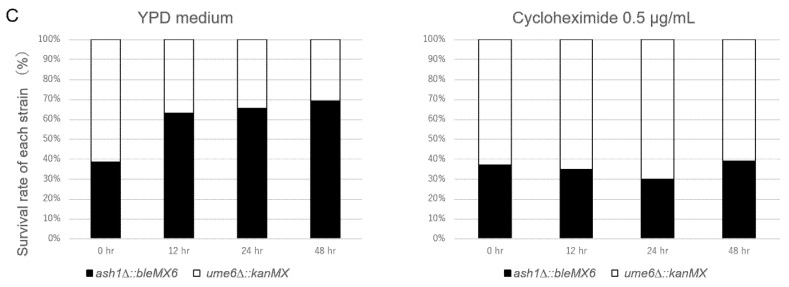
The ρ^+^ cells of the *ume6*∆ mutant are more tolerant to fluconazole and cycloheximide than those of the *ash1*∆ and *gat3*∆ mutants in the cocultivation assays. (**A**) Changes in the survival rates of each strain after cocultivation of the *ume6*∆꞉꞉*bleMX6 and ash1*∆꞉꞉*KanMX* strains in YPG medium with (right) and without (left) of 0.5 μg/mL cycloheximide. (**B**) Rates of viable cells of each strain after cocultivation of the *gat3*∆꞉꞉*bleMX6 and ume6*∆꞉꞉*KanMX* strains using YPG medium with (right) and without (left) 30 μg/mL fluconazole. (**C**) Changes in the survival rates of each strain after cocultivation of the *ash1*∆꞉꞉*bleMX6 and ume6*∆꞉꞉*KanMX* strains in YPD medium with (right) and without (left) of 0.5 μg/mL cycloheximide.

In the spot dilution assay above, the ρ^+^ cells of the *ume6*∆ mutant displayed slightly more tolerance to fluconazole and cycloheximide in YPG plates than the ρ^+^ cells of the wild-type, *pdr3*∆, *ash1*∆, and *gat3*∆ strains (Figure 1). To detect a slight difference in drug resistance between two mutant strains, we previously used a cocultivation assay [32]. Therefore, we further investigated whether the ρ^+^ cells of the *ume6*∆ mutant confer fluconazole and cycloheximide tolerance in a cocultivation assay using YPG medium. The *ash1*∆ and *gat3*∆ mutants displayed an equal tolerance to fluconazole and cycloheximide compared to the wild-type strain in the spot dilution assay (Figure 1), and thereby, the *ash1*∆ and *gat3*∆ mutants were used as controls for the *ume6*∆ mutant in the cocultivation assay. ρ^+^ cells of two mutant strains, *ash1*∆꞉꞉*kanMX* and *ume6*∆꞉꞉*bleMX6,* were cocultivated in the presence and absence of 0.5 μg/mL cycloheximide in YPG medium. The number of viable cells in each mutant strain in the coculture was estimated by spreading the cells on YPD plates containing G418 or Zeocin. We found that the ρ^+^ cells of the *ume6*∆꞉꞉*bleMX6* strain were eliminated from the coculture over time in YPG medium without cycloheximide but accounted for a large percentage of the viable cells in the coculture over time in YPG medium containing 0.5 μg/mL cycloheximide (*p* < 0.05) (Figure 2A). Similarly, the ρ^+^ cells of the *ume6*∆꞉꞉*KanMX* strain were eliminated from the cocultures of the *ume6*∆꞉꞉*KanMX* and *ash1*∆꞉꞉*bleMX6* strains in YPG medium over time in the absence of cycloheximide but selected in the presence of 0.5 μg/mL cycloheximide (data not shown). In addition, the ρ^+^ cells of the *ume6*∆꞉꞉*KanMX* strain were eliminated from the coculture over time in the absence of fluconazole but not in the presence of fluconazole when cocultivated with *gat3*∆꞉꞉*bleMX6* in YPG medium with or without 30 μg/mL fluconazole (Figure 2B). Furthermore, similar results were observed for the ρ^+^ cells of the *gat3*∆꞉꞉*KanMX* and *ume6*∆꞉꞉*bleMX6* mutants cocultivated in YPG medium with or without 30 μg/mL fluconazole (data not shown).

We next examined whether the higher cycloheximide and fluconazole resistance in *ume6*∆ is dependent on the carbon source used in the growth medium. Rather than using the coculture grown in YPG medium, the survival rate of each strain using the coculture grown in YPD medium was estimated in the same way. Consequently, the ρ^+^ cells of the *ume6*∆꞉꞉*KanMX* strain were eliminated from the coculture over time in the absence of cycloheximide but not in the presence of 0.5 μg/mL cycloheximide when cocultivated with the *ash1*∆꞉꞉*bleMX6* mutant in YPD medium (*p* < 0.05) (Figure 2C). Furthermore, similar results were observed for the ρ^+^ cells of the *gat3*∆꞉꞉*KanMX* and *ume6*∆꞉꞉*bleMX6* strains cocultivated in YPD medium with or without 30 μg/mL fluconazole (or 0.5 μg/mL cycloheximide) (data not shown). These results indicate that, in contrast to previous reports, *UME6* acts as a silencer rather than an enhancer of drug resistance in ρ^+^ cells of *S. cerevisiae* independent of the carbon source used in the growth medium.

### 3.2. UME6 Is Required for the Suppression of Basal ABC Transporter mRNA Levels

**Figure 3:**
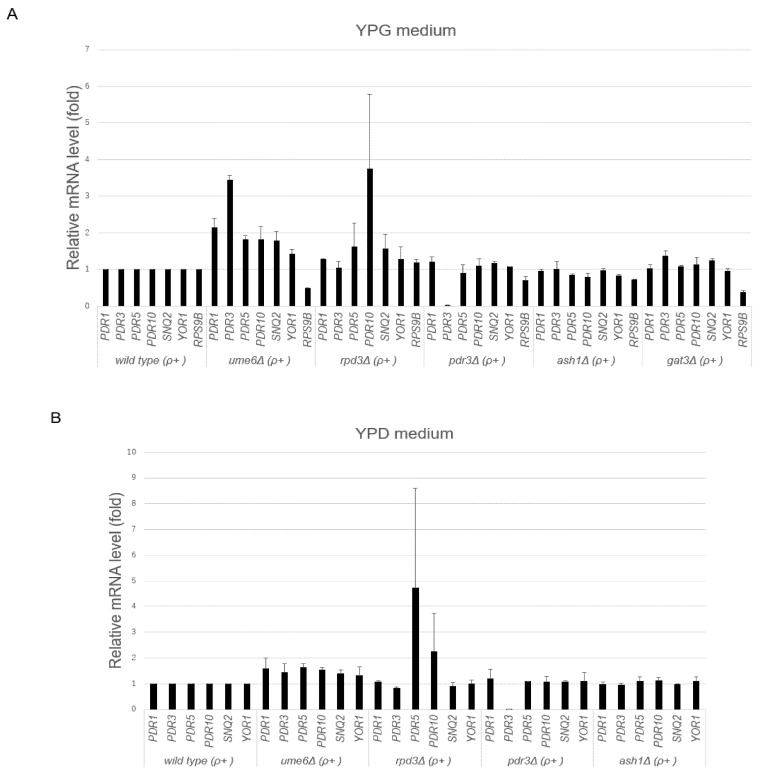
Transcription levels of ABC transporters and their regulators in ρ^+^ cells of the wild-type and mutant strains in the logarithmic growth phase. (**A**) ρ^+^ cells of the wild-type, *ume6*∆, *rpd3*∆, *pdr3*∆, *ash1*∆, and *gat3*∆ strains were grown to the log phase in YPG medium. Relative *PDR1, PDR3, PDR5, PDR10, SNQ2, YOR1,* and *RPS9B* mRNA levels were determined by qRT-PCR. (**B**) Relative *PDR1, PDR3, PDR5, PDR10, SNQ2,* and *YOR1* mRNA levels in ρ^+^ cells of the wild-type, *ume6*∆, *rpd3*∆, *pdr3*∆, and *ash1*∆ strains grown to the log phase in YPD medium were determined by qRT-PCR.

Decreased drug resistance was found in the ρ^+^ cells of the *rpd3*∆ strain in the spot dilution assay, while increased drug resistance in the ρ^+^ cells of the *ume6*∆ strain was found in the spot dilution and cocultivation assays. These results suggested that the transcriptional levels of ABC transporters such as *PDR5* are reduced in the ρ^+^ cells of the *rpd3*∆ mutant and increased in the ρ^+^ cells of the *ume6*∆ mutant. Thus, we investigated the mRNA levels of ABC transporters *PDR5, PDR10, SNQ2,* and *YOR1* and their transcriptional regulators *PDR1* and *PDR3* in the ρ^+^ cells of the wild-type, *ume6*∆, *rpd3*∆, *pdr3*∆, *ash1*∆, and *gat3*∆ strains grown in YPG medium to the logarithmic phase by real-time RT-PCR*. PDR1, PDR3, PDR5, SNQ2,* and *YOR1* but not *PDR10* expression was significantly induced in the *ume6*∆ strain compared with the wild-type strain (*p* < 0.05), while *RPS9B* expression, used as a control, was downregulated in the *ume6*∆ strain compared with the wild-type strain (Figure 3A). The transcriptional induction of these ABC transporters and their transcriptional regulators can explain the increased tolerance of the *ume6*∆ mutant to fluconazole and cycloheximide in the spot dilution and cocultivation assays using YPG medium. In contrast, *PDR1* expression, but not *PDR3*, *PDR5*, *PDR10, SNQ2*, or *YOR1* expression, was significantly more induced in the *rpd3*∆ strain than in the wild-type strain (*p* < 0.05) (Figure 3A). The lack of significant changes in expression of the ABC transporters, including *PDR5*, between the wild-type and *rpd3*∆ strains cannot explain the high susceptibility of the *rpd3*∆ mutant to fluconazole and cycloheximide in the spot dilution assays using YPG plates (Figure 1 and Figure 3A). Therefore, the susceptibility of the *rpd3*∆ mutant to fluconazole and cycloheximide may result from impaired Hsp90-dependent antifungal drug resistance and ER-to-Golgi trafficking of Pdr5 in YPG medium and not from *PDR5* transcriptional reduction [34,45]. In contrast to the *ume6*∆ mutant, the *PDR5* and *YOR1* mRNA levels were significantly reduced in the *ash1*∆ mutant compared with the wild-type strain (*p* < 0.05) (Figure 3A). 

Next, we investigated the mRNA levels of the ABC transporters *PDR5, PDR10, SNQ2,* and *YOR1* and their transcriptional regulators *PDR1* and *PDR3* in ρ^+^ cells of the wild-type, *ume6*∆, *rpd3*∆, *pdr3*∆, and *ash1*∆ strains grown to the logarithmic phase in YPD medium in the same way. In contrast to the results obtained by real-time RT-PCR using YPG medium, only the basal *PDR5* and *PDR10* mRNA levels were significantly upregulated in the *ume6*∆ strain compared with the wild-type strain in YPD medium (*p* < 0.05) (Figure 3B). In addition, the degrees of upregulation of basal transcription of ABC transporters and their regulators in the *ume6*∆ strain are relatively lower in YPD medium than in YPG medium (Figure 3A,B). However, the increased drug resistance of the *ume6*∆ mutant shown in the cocultivation assays using YPD can be explained by the induction in the *PDR5* mRNA level. In contrast, there was no significant difference in mRNA levels of the ABC transporters and their regulators between ρ^+^ cells of the wild-type and *rpd3*∆ strains grown in YPD medium (*p* > 0.05) (Figure 3B). Therefore, the susceptibility of the *rpd3*∆ mutant to fluconazole and cycloheximide found in the spot dilution assays using YPD plates may result from impaired Hsp90-dependent antifungal drug resistance but not from the reduced *PDR5* mRNA level [34]. In addition, different from the results obtained by real-time RT-PCR using YPG medium, no significant changes in expression of the ABC transporters and their regulators were found between the wild-type and the *ash1*∆ strains in YPD medium (Figure 3B).

**Figure 4:**
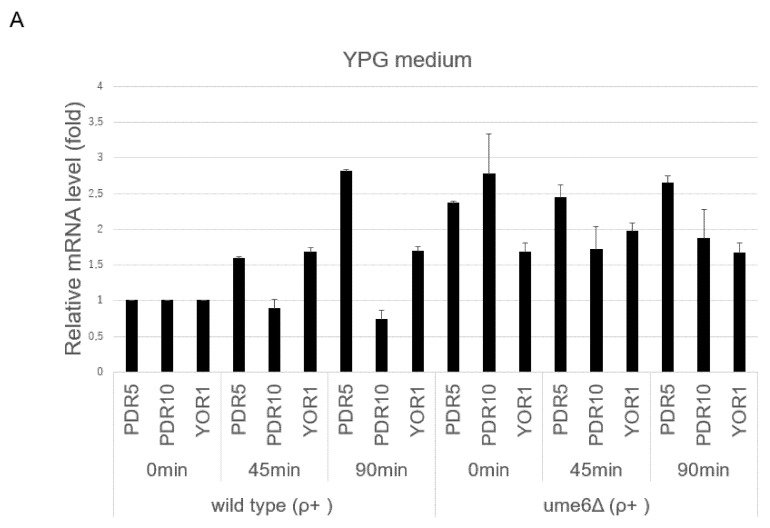

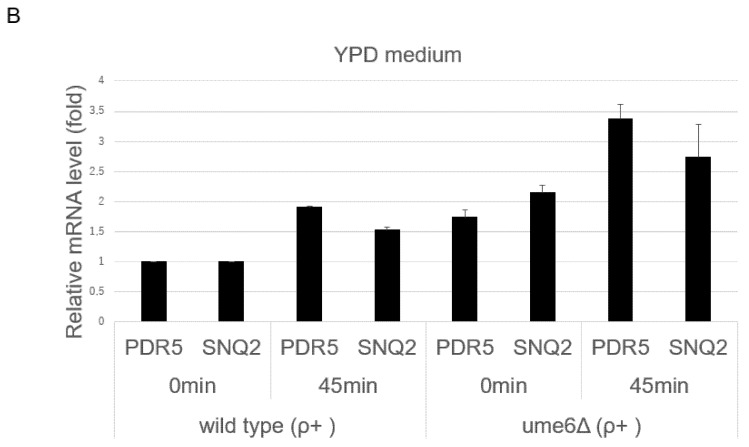
Changes in the transcriptional expression *of PDR5, PDR10, YOR1,* and *SNQ2* in the ρ^+^ cells of the wild-type and *ume6*∆ strains in YPG and YPD media after the addition of cycloheximide. (**A**) The relative fold levels *of PDR5, PDR10,* and *YOR1* mRNA in the ρ^+^ cells of the wild-type and *ume6*∆ strains after exposure to cycloheximide (0.2 μg/mL) for 45 min and 90 min in YPG medium were quantified by qRT-PCR. (**B**) The relative fold levels of *PDR5* and *SNQ2* mRNA in the ρ^+^ cells of the wild-type and *ume6*∆ strains at 45 min after the addition of cycloheximide (0.2 μg/mL) in YPD medium were quantified by qRT-PCR.

Furthermore, we investigated whether transcriptional activation of the ABC transporters following cycloheximide exposure occurs in the ρ^+^ cells of the *ume6*∆ mutant in YPG and YPD media using real-time RT-PCR. In YPG and YPD media, the ABC transporter mRNA levels in the *ume6*∆ strain at 0 min were higher than those in the wild-type strain at 0 min (*p* < 0.05), which is consistent with the results in Figure 3A,B (Figure 4A,B). This also suggested that *UME6* is required for the repression of basal transcription of the ABC transporters, including *PDR5*, in ρ^+^ cells, independent of the media used. In addition, the degree of upregulation of *PDR5* transcription in the ume6∆ strain at 0 min was relatively lower in YPD medium than in YPG medium, which is consistent with the results in Figure 3A,B (Figure 4A,B). On the other hand, the ABC transporter mRNA levels, except for *PDR10*, in the wild-type strain were significantly higher at 45 min than at 0 min in YPG and YPD media (*p* < 0.05) (Figure 4A,B). In contrast, the *PDR5*, *PDR10*, and *YOR1* mRNA levels in the u*me6*∆ strain were not different between 0 min and 45 min in YPG medium (*p* > 0.05) (Figure 4A). In YPD medium, the *PDR5*, but not *SNQ2*, mRNA levels in the u*me6*∆ strain were significantly higher at 45 min than at 0 min (*p* < 0.05) (Figure 4B). These results suggested that *UME6* is required for the intact induction of *PDR5* and *YOR1* transcription in ρ^+^ cells after drug exposure in YPG medium but only for the intact induction of *SNQ2* in YPD medium.

## 4. Discussion

In this report, we have shown that *UME6* suppresses the basal transcription levels of the ABC transporters, including *PDR5*, and drug resistance in the ρ^+^ cells of *S. cerevisiae* independent of the carbon source used in the growth medium while *RPD3* does not interfere with basal *PDR5* transcription but contributes to drug resistance. The results found for *RPD3* in this study were consistent with previous reports [34]. However, it has not been previously reported that *UME6* suppresses basal *PDR5* mRNA levels and drug resistance in the ρ^+^ cells of *S. cerevisiae*. ρ^+^ cells of the *ume6*∆ strain conferred more resistance to fluconazole and cycloheximide than those of the wild-type strain in the spot dilution assay using YPG plates but not using YPD plates. In addition, although the ρ^+^ cells of the *ume6*∆ mutant were eliminated from the coculture over time in both YPG and YPD media without cycloheximide, these cells accounted for the majority of the coculture over time in YPG medium containing 0.5 μg/mL cycloheximide (Figure 2A) while maintaining a constant survival rate over time in YPD medium containing 0.5 μg/mL cycloheximide (Figure 2C). These results can be explained by the appearance of ρ^0^ cells within the ρ^+^ cells in the wild-type and *ume6*∆ strains in YPD medium because ρ^0^ cells of the *ume6*∆ mutant are more sensitive to fluconazole and cycloheximide than the ρ^0^ cells of the wild-type strain [32]. Inverse transcriptional regulation of *PDR5* is reported between the ρ^0^ cells of the *ume6*∆ and *ash1*∆ mutants, in which the *PDR3* and *PDR5* mRNA levels are downregulated in the ρ^0^ cells of the *ume6*∆ mutant but upregulated in the *ash1*∆ mutant [32]. In addition, the fold expression levels of most of the ABC transporters in the *ume6*∆ mutant compared to the wild-type strain were downregulated in YPD medium compared to YPG medium, while those in the *ash1*∆ mutant compared to the wild-type strain were upregulated in YPD medium compared to YPG medium (Figure 3A,B and Figure 4A,B). Thus, this inverse transcriptional correlation in the ρ^+^ cells of the *ume6*∆ and *ash1*∆ mutants between YPD and YPG media can also be explained by the occurrence of ρ^0^ cells within the ρ^+^ cells of the *ume6*∆ and *ash1*∆ mutants in YPD medium. As histone deacetylation leads to transcriptional repression and activation, Ume6 may serve as a silencer of *PDR5* expression in ρ^+^ cells and an enhancer of *PDR5* expression in ρ^0^ cells [27,47]. Furthermore, the inverse transcriptional regulation of the *PDR5* between the *ume6*∆ and *ash1*∆ mutants independent of ρ^+^ and ρ^0^ cells may be caused by exclusive and elevated recruitment of the Rpd3L complex to the target regions by Ume6 in the *ash1*∆ mutant. Considering the above, due to the relatively low suppression levels of basal *PDR5* transcription and drug resistance by *UME6* in YPD medium, which could be led by the occurrence of ρ^0^ cells within ρ^+^ cells, the roles of *UME6* as a negative regulator of basal *PDR5* transcription and drug resistance in ρ^+^ cells may have not been reported. In such cases, a co-cultivation assay but not a spot dilution assay may be useful for detecting a slight difference in drug resistance. 

**Figure 5:**
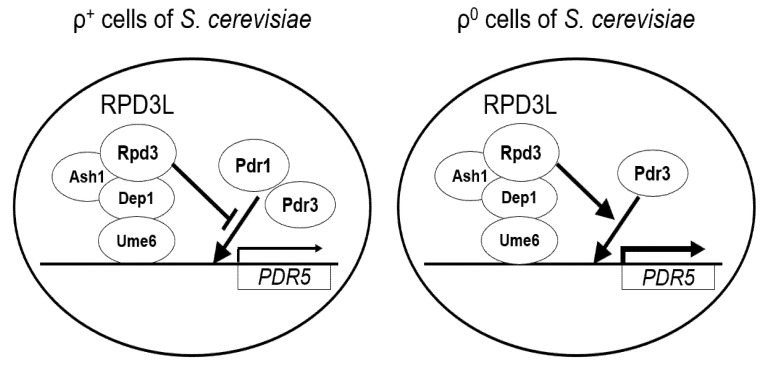
A simplified diagram of the regulation of basal transcription of *PDR5* by Ume6 and Rpd3.

It is currently unknown how Ume6 suppresses the basal transcriptional expression of *PDR5* and drug resistance in ρ^+^ cells and inversely enhances basal *PDR5* expression and drug resistance in ρ^0^ cells [32]. As Ume6 binds to the promoter region of the ABC transporters such as *PDR5*, *PDR10*, and *YOR1* in ρ^+^ cells, Ume6 may directly mediate the repression of the ABC transporters by recruiting the Rpd3L and Isw2 chromatin remodeling complexes (Figure 5) [48]. Furthermore, suppression of the ABC transporter transcription and drug resistance by Ume6 in ρ^+^ cells may be indirectly caused by changes in the expression of the regulators of ABC transporters, such as Pdr1 and Pdr3. 

The transcriptional mediator complex serves as a link between sequence-specific transcription factors and the RNA polymerase II machinery [49]. There are two different forms in the transcriptional mediator complex in *S. cerevisiae*: the core Mediator and L-Mediator. The L-Mediator complex contains the core Mediator complex and the Cdk8 subcomplex. The Cdk8 subcomplex consists of Med12 (Srb8), Med13 (Srb9), the cyclin-dependent kinase Cdk8 (Srb10), and cyclin C (Srb11). The Cdk8 subcomplex has both a negative and a positive role in gene transcription [49,50,51]. Pdr1 and Pdr3 can bind to the KIX domain of a mediator subunit called Med15/Gal11 of the core Mediator and L-Mediator [17]. Deletion of Med12 from the Cdk8 complex completely suppresses the induction of *PDR5* expression in ρ^0^ cells but not in ρ^+^ cells, indicating a difference in the regulatory machinery of *PDR5* transcription between ρ^+^ and ρ^0^ cells [52]. This difference may be relevant to the difference in transcriptional regulation of *PDR5* by Ume6 between ρ^0^ and ρ^+^ cells. 

This study indicated that *UME6* is required for the intact enhancement of *PDR5* and *YOR1* transcription after cycloheximide exposure in YPG medium and for intact cycloheximide-induced transcription of *SNQ2*, but not *PDR5*, in YPD medium. This result suggested that basal transcriptional repression of the ABC transporters, including *PDR5*, by *UME6* is lifted in response to drug exposure in YPG medium. However, it is unknown why the intact induction of *PDR5*, but not *SNQ2*, in the *ume6*∆ mutant occurs after cycloheximide exposure in YPD medium (Figure 4B). This may result from that cycloheximide can distort measurements of mRNA levels [53]. However, which of upregulation in basal *PDR5* transcription or intact drug-induced *PDR5* upregulation in the *ume6*∆ mutant is more effective for drug resistance in YPD medium? Despite intact cycloheximide-induced transcription of *PDR5* in the *ume6*∆ strain in YPD medium, there was no difference in cycloheximide resistance between the ρ^+^ cells of the wild-type and *ume6*∆ strains in the spot dilution assay using YPD plates, while a little difference in the cocultivation assay using YPD plates. In addition, upregulation of basal *PDR5* transcription levels in the *ume6*∆ strain compared to the wild-type strain was relatively lower in YPD medium than in YPG medium. Therefore, the upregulation of basal *PDR5* transcription in the *ume6*∆ strain may be more important for drug resistance than intact transcriptional upregulation of *PDR5* in the *ume6*∆ strain following cycloheximide exposure. 

## 5. Conclusions

This study investigated the roles of *RPD3* and *UME6* in basal and drug-induced transcription of the ABC transporters, including *PDR5*, and PDR in the ρ^+^ cells of *S. cerevisiae*. Using spot dilution and cocultivation assays and YPG and YPD media, we have shown that *RPD3* contributes to drug resistance in ρ^+^ cells, while in contrast to the previous reports, *UME6* contributes to the suppression of drug resistance in ρ^+^ cells, independent of the carbon source used in the growth medium. In addition, using a real-time PCR assay, we have shown that *RPD3* does not interfere with the basal *PDR5* mRNA level in ρ^+^ cells, while *UME6* is involved in the suppression of basal transcription of some ABC transporters, including *PDR5*, in ρ^+^ cells regardless of the carbon source utilized. We have also described that *UME6* contributes to an intact transcriptional enhancement of *PDR5* in cycloheximide-exposed ρ^+^ cells in YPG medium but not in YPD medium. This and our previous work will provide useful knowledge on yeast multidrug resistance via the transcriptional regulation of efflux genes by *UME6* in ρ^+^ and ρ^0^ cells.

## Data Availability

All data generated or analysed during this study are included in this published article.

## Supplementary Materials

The following are available online at https://www.mdpi.com/article/10.3390/microorganisms10030601/s1, Table S1: Primers used for qRT-PCR, Figure S1: Cycloheximide resistance of ρ+ cells of the wild-type and its derivative strains.
