# Supplementary material for: UME6 Is Involved in the Suppression of Basal Transcription of ABC Transporters and Drug Resistance in the ρ+ Cells of Saccharomyces cerevisiae
Source: Microorganisms. 2022 Mar 10;10(3):601. doi: 10.3390/microorganisms10030601 (PMC8953597; doi:10.3390/microorganisms10030601)
Supplement: Supplementary file 1 [file microorganisms-10-00601-s001.zip › Figure S1 for microorganisms.pptx]

## Slide 1
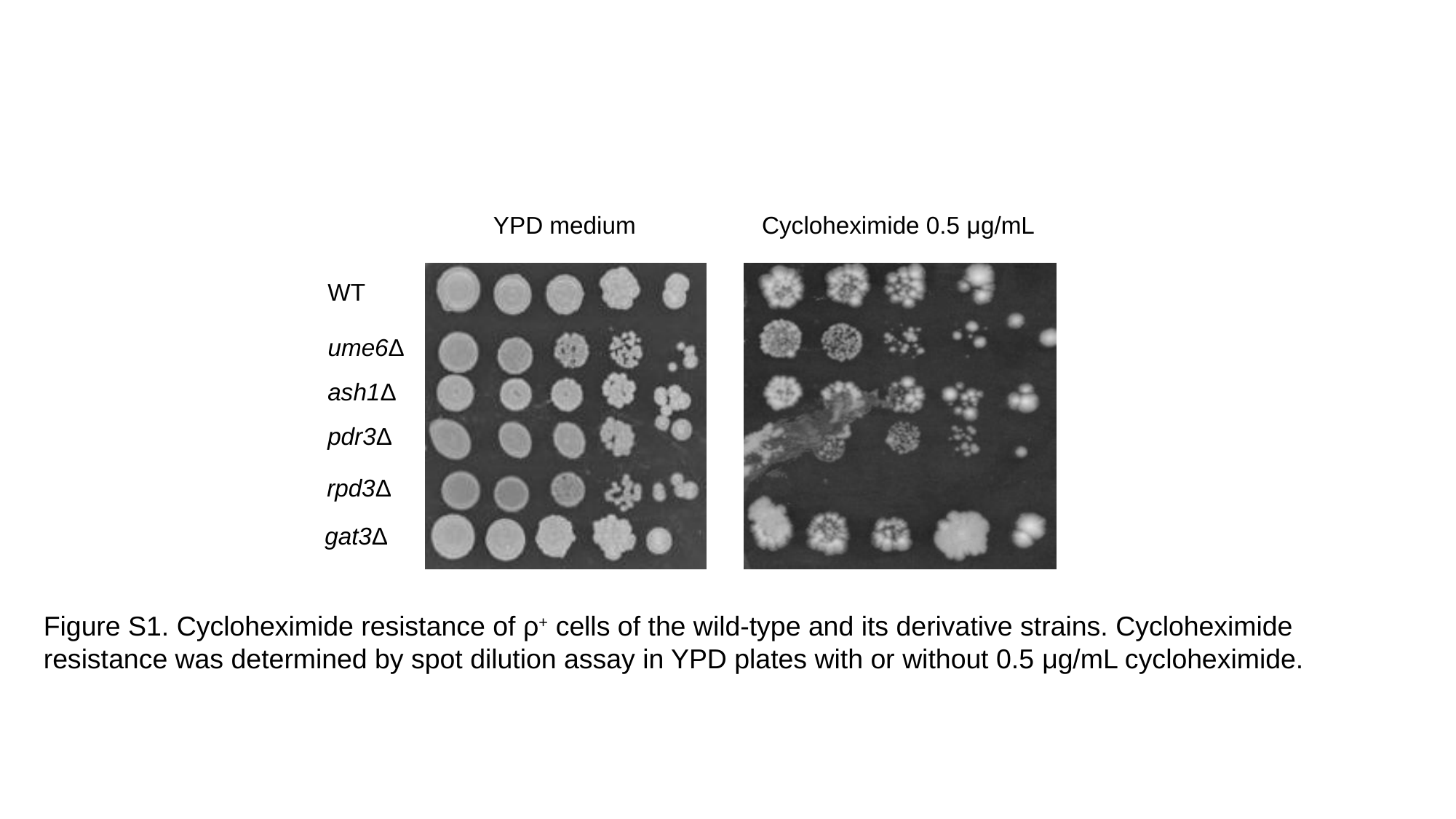

YPD medium
Cycloheximide 0.5 μg/mL
WT
ume6Δ
ash1Δ
pdr3Δ
rpd3Δ
gat3Δ
Figure S1. Cycloheximide resistance of ρ+ cells of the wild-type and its derivative strains. Cycloheximide resistance was determined by spot dilution assay in YPD plates with or without 0.5 μg/mL cycloheximide.
